# Recent Advances in Plastic Waste Biodegradation: Microorganisms, Enzymes, Invertebrates, and Evidence Standards

**DOI:** 10.3390/biology15191726

**Published:** 2026-10-01

**Authors:** Key-Seem Phang, Chou-Min Chong, Yan Chen, Jiun-Yan Loh

**Affiliations:** 1 Faculty of Applied Sciences, UCSI University, No. 1, Jalan UCSI, UCSI Heights, Taman Connaught, Cheras, Kuala Lumpur 56000, Malaysia; keyphang@hotmail.com; 2 Laboratory of Sustainable Aquaculture (AquaLab), International Institute of Aquaculture and Aquatic Sciences (I-AQUAS), Universiti Putra Malaysia, Port Dickson 71050, Negeri Sembilan, Malaysia; choumin@upm.edu.my; 3 Yazhou Bay Innovation Institute, Hainan Tropical Ocean University, Sanya 572024, China; cy969398hn@126.com; 4 Tropical Futures Institute, James Cook University (Singapore), 149 Sims Drive, Singapore 387380, Singapore

**Keywords:** biodegradation, microorganisms, microplastics, plastic-eating invertebrates, synthetic polymers

## Abstract

The uncontrolled and rapidly increasing use of synthetic polymers has created persistent environmental contamination. Millions of tonnes of plastic waste accumulate annually, and fragmentation generates microplastics that threaten terrestrial and aquatic ecosystems and may affect human health. Biodegradation has therefore attracted attention as a complementary strategy for plastic-waste management. Reported biological agents include bacteria and fungi, their enzymes, and plastic-consuming invertebrates such as mealworms and waxworms. This review synthesizes evidence on microbial, enzymatic, and invertebrate-mediated degradation of common synthetic polymers and examines the physicochemical changes used to assess degradation, including changes in molecular weight, surface morphology, hydrophilicity, functional groups, and mineralization products. It also considers biodegradable polymers derived wholly or partly from renewable feedstocks as alternatives to conventional petroleum-based plastics. Emphasis is placed on the evidence required to demonstrate genuine biodegradation, because surface damage, biofilm formation, or mass loss alone can be misleading. Current research gaps, methodological limitations, and priorities for standardized, reproducible testing are discussed to support the development of credible biological approaches to plastic-waste reduction.

## Simple Summary

Plastic waste is a growing global problem, with hundreds of millions of tonnes produced annually and substantial quantities entering landfills, rivers, and oceans. Persistent plastics fragment into microplastics, which have been detected in food, water, and the human body, raising ecological and health concerns. Biodegradation offers a promising response by using microorganisms, enzymes, and invertebrates such as mealworms and waxworms to break down plastics. This review evaluates biological degradation pathways, methods used to verify degradation, and biodegradable alternatives derived from renewable materials. It also identifies key research gaps, particularly the lack of internationally harmonized standards for reliably assessing plastic biodegradability.

## 1. Introduction

Plastics are synthetic or semi-synthetic polymeric materials that can be shaped by heating and are produced by chemically linking monomers into macromolecular chains [1]. Technological advances have enabled their widespread use in packaging, construction, furniture, automotive manufacturing, aerospace applications, consumer goods, and medical products. Polystyrene (PS), polyethylene (PE), polyurethane (PUR), and polypropylene (PP) are among the most widely used plastics and together account for up to 80% of global plastic consumption [2,3]. Since the 20th century, plastics have transformed modern life because their mechanical and barrier properties provide durability, corrosion resistance, low density, chemical inertness, and low production costs.

These properties have driven extensive, and often poorly controlled, production and consumption. Approximately 500 million tonnes of plastic are used annually across diverse applications [4,5]. Global plastic production has grown steadily for decades and reached approximately 431 million tonnes in 2024, roughly a sevenfold increase since the 1980s [6]. Large quantities consequently enter landfills and aquatic environments, where they may persist for decades and contribute to long-term pollution [7,8].

Biodegradation has been proposed as one component of a broader strategy for managing synthetic polymers. It is a natural process in which microorganisms, principally fungi and bacteria, transform polymeric materials through enzymatic reactions [9,10]. Extracellular enzymes first cleave long polymer chains into lower-molecular-weight fragments, oligomers, and monomers that may subsequently be assimilated and mineralized to CO_2_, H_2_O, and biomass under aerobic conditions, or to CH_4_ and other products under anaerobic conditions. Because complete mineralization is distinct from mere fragmentation, claims of biodegradation require evidence beyond surface deterioration or mass loss. Research on fungal and bacterial degradation of synthetic polymers has therefore expanded substantially [11,12,13,14,15,16,17].

This review examines microbial and enzymatic plastic biodegradation, invertebrate-associated processes, and the factors that influence biodegradability. It critically evaluates commonly used synthetic plastics and the evidence reported for their degradation. Specifically, the review aims to (1) describe plastic pollution and its potential implications for human health; (2) explain the mechanisms through which microorganisms and enzymes interact with plastic polymers and their blends; (3) synthesize recent research on microbial, invertebrate-mediated, and biopolymer-based approaches; (4) evaluate methods used to measure and verify polymer degradation; and (5) identify future challenges in plastic-waste management.

Unlike prior reviews in this area that largely catalogue reported degradation results, this review applies a consistent evidentiary standard across that literature: every study discussed is classified by what it demonstrated, e.g., surface alteration, depolymerization, or mineralization, rather than by whether biodegradation was claimed. Section 5.8 sets out why this distinction matters methodologically, and Tables 1 and 2 apply it directly, so a reader can see briefly which findings rest on isotope- or respirometry-confirmed mineralization and which rest on weight loss or surface imaging alone.

### Search Strategy

Literature for this review was collected from the Web of Science, Scopus, and Google Scholar databases. Searches combined the terms “plastic biodegradation,” “polymer degradation,” “biodegradable plastics,” and “microplastic” with the name of each polymer covered (polyethylene, polystyrene, polyvinyl chloride, polyethylene terephthalate, polypropylene, and polyurethane) and with the genus or species names of the microorganisms and invertebrates discussed, such as *Tenebrio molitor*, *Galleria mellonella*, *Zophobas atratus*, *Pseudomonas*, and *Rhodococcus*. The search prioritized studies from the past decade, though a small number of older papers were kept if they first established a mechanism or method still cited in current work. Only peer-reviewed primary research and review articles in English were considered. Conference abstracts without an accompanying full paper, non-peer-reviewed sources, and studies describing only mechanical or thermal degradation with no microbial, enzymatic, or invertebrate involvement were excluded.

## 2. Plastic Polymer Pollution and Degradation

The rapid growth of plastic production has led to the accumulation of large quantities of persistent waste in the environment, creating a substantial challenge for waste-management systems [18]. Most commercially available plastics are synthetic materials composed of high-molecular-weight polymer chains with strong chemical bonds. Their formulations and manufacturing processes confer durability but also make them resistant to degradation under natural conditions [19]. Consequently, plastics can remain in terrestrial and marine environments for decades, while reliable estimates of their biodegradation rates remain limited [20].

Additives such as flame retardants, stabilizers, and lubricants may further inhibit oxidation and degradation, contributing to the persistence of plastic products under diverse environmental conditions. Plastic waste in landfills and marine environments is exposed to sunlight, mechanical abrasion, temperature fluctuations, and other weathering processes that can cause cracking and fragmentation. These processes generate microplastics, commonly defined as plastic particles smaller than 5 mm, including particles in the micrometer range. Plastic debris and microplastics threaten terrestrial and aquatic organisms through ingestion, entanglement, and associated effects such as intestinal obstruction [21,22,23].

The environmental and health implications of plastic pollution have received considerable public and scientific attention [24,25]. Humans are exposed to microplastics and nanoplastics through multiple routes, including food, drinking water, and inhalation [26,27,28]. Their small size also makes them available to marine organisms and facilitates transfer through food webs; particles have been detected in seafood such as oysters, crabs, and fish [29,30]. In a pilot study, Braun et al. [31] identified ten types of microplastics, including polyethylene, in placental and meconium samples from two mother–infant pairs, with particles measuring up to 50 µm. Ragusa et al. [32] likewise detected microplastic fragments, including PP, in placentas from consenting women with uncomplicated pregnancies using Raman microspectroscopy. Subsequent biomonitoring studies have reported plastic particles in human blood and lung tissue [33,34]. These findings confirm human exposure but do not by themselves establish clinical harm. Experimental and epidemiological evidence has raised concerns about inflammation, oxidative stress, reproductive effects, metabolic disruption, and carcinogenic potential, although causal relationships and dose–response thresholds in humans remain uncertain [35,36,37,38,39,40,41,42]. Conventional management approaches—including recycling, landfilling, and incineration—also have limitations, such as incomplete recovery, greenhouse-gas emissions, land use, and the release of hazardous compounds [36,37]. These constraints have stimulated the development of complementary biotechnological approaches [38,39].

## 3. Polymer Biodegradation Mechanism

Biodegradation involves the biological transformation of materials by organisms such as bacteria, fungi, and algae. For plastics, it may include reductions in molecular weight and mechanical strength, changes in surface properties, depolymerization, assimilation, and, ultimately, mineralization [40]. Complete mineralization converts polymer-derived carbon into CO_2_, H_2_O, and biomass under aerobic conditions, or into CH_4_ and other products under anaerobic conditions, thereby returning carbon to biogeochemical cycles [41]. As illustrated in Figure 1, polymer biodegradation is commonly described in four overlapping stages:

### 3.1. Colonization

Most polymer surfaces are hydrophobic, and their molecules have high molecular weights, microbial attachment and colonization are generally considered the initial stages of biodegradation. Cells adhere to the polymer surface and may form biofilms, while microbial activity and extracellular products introduce hydrophilic groups that alter the surface’s physical and chemical properties [43,44,45].

### 3.2. Depolymerization

Plastic polymers are generally too large to cross microbial cell membranes. They must therefore undergo biodeterioration and depolymerization into smaller fragments that can be transported into cells and metabolized [20]. Microorganisms and other organisms promote this process through physical action and the secretion of extracellular enzymes [46]. The rate of deterioration and depolymerization depends on the polymer’s properties, environmental conditions, and the activity of organisms colonizing its surface. Cleavage and oxidation of long hydrocarbon chains reduce molecular weight and generate oligomers, dimers, and monomers with new functional groups [47].

### 3.3. Assimilation

Microbial receptors and transport systems recognize sufficiently small oligomers, dimers, or monomers and facilitate their uptake across the cell membrane. Within the cell, these compounds enter metabolic pathways that generate energy, biomass, and primary or secondary metabolites [41]. Enzymatic biodegradation begins with enzyme binding to the polymer substrate, followed by hydrolytic or oxidative chain cleavage. Extracellular depolymerases generate smaller products, whereas intracellular enzymes further metabolize assimilated compounds. Depending on the polymer and organism, these intermediates may be converted into fatty acids, organic acids, or other metabolites [48].

### 3.4. Mineralization

During mineralization, assimilated intermediates are converted into inorganic end products and biomass. Under aerobic conditions, the principal products are CO_2_ and H_2_O, under anaerobic conditions, CH_4_ and other reduced products may also form [49]. Polymer biodegradation is strongly influenced by molecular weight, hydrophobicity, tacticity, crystallinity, functional groups, additives, and environmental conditions such as temperature, moisture, oxygen availability, and nutrient supply [38,41].

## 4. Biotechnology in Polymer Biodegradation

The physicochemical properties of many conventional plastics include high hydrophobicity, low bioavailability, high crystallinity, and limited reactive functional group contribute to their extremely slow degradation under natural conditions. Nevertheless, advances in biotechnology have identified organisms, enzymes, and pretreatment strategies that can transform selected polymers into more degradable products [50,51]. These studies continue to expand the range of microbial taxa and catalytic systems with potential applications in plastic depolymerization and valorization.

### 4.1. Microbial Biodegradation

Microbial biodegradation uses the metabolic and enzymatic capabilities of microorganisms to transform synthetic polymers. Hydrolytic and oxidative enzymes, including cutinases, lipases, esterases, peroxidases, and proteases, can cleave polymer bonds or introduce functional groups that increase surface hydrophilicity and accessibility [40,52]. Enzyme production and substrate specificity vary among species and strains, and no single microorganism is expected to degrade all polymer types efficiently. Bacteria and fungi that colonize hydrophobic surfaces and produce complementary enzyme systems are therefore important candidates for polymer-degradation studies [52,53,54]. This asymmetry in enzymatic progress is not incidental. PET, PUR, PLA, and PHA all contain hydrolyzable backbone linkages—ester or urethane bonds that hydrolytic enzymes such as cutinases, esterases, and ureases can invade directly with a single well-defined bond-cleavage mechanism that protein engineering can systematically optimize. PE, PP, and PS, by contrast, are built on a continuous carbon–carbon backbone with no such hydrolysable target; degrading them requires oxidative enzymes such as laccases, peroxidases, alkane hydroxylases, and cytochrome P450s that attack an inert bond non-specifically, typically only after abiotic weathering has already introduced oxygen into the polymer. This is why enzyme engineering for PET has produced near-complete depolymerization in hours [6,55], while the PE- and PS-degrading enzymes discussed below still mostly produce surface-level evidence over weeks to months. The distinction is mechanistic, not a matter of research effort: rational engineering has a defined active site to improve hydrolytic enzymes, and no equivalent single target for oxidative ones. Recent enzyme-engineering studies have markedly accelerated the depolymerization of poly(ethylene terephthalate) (PET). FAST-PETase acts on several untreated post-consumer PET products, whereas TurboPETase achieved nearly complete depolymerization within 8 h at a solid loading of 200 g/kg [55,56]. These advances demonstrate the value of combining natural enzyme discovery with protein engineering, although performance under laboratory conditions does not necessarily predict environmental degradation.

Researchers have evaluated numerous pure microbial strains for their ability to degrade plastic polymers [13]. Members of the metabolically diverse genus *Pseudomonas*, which occur widely in terrestrial and marine environments, have received particular attention because they can colonize and transform hydrocarbons and hydrophobic plastics with varying efficiencies [57,58,59]. Tribedi and Sil [43], for example, reported enhanced biofilm formation by *Pseudomonas* sp. AKS2 on low-density polyethylene (LDPE) film, reaching approximately 300 mg after one month of incubation. Another study reported that a pure *Pseudomonas* isolate converted 28.6% of low-molecular-weight polyethylene into CO_2_ within two months [60]. *Pseudomonas* strains have also been investigated for the degradation of high-impact polystyrene (HIPS). Mohan et al. [61] observed that unclassified *Pseudomonas* strains remained viable for two weeks with HIPS film as the sole carbon and energy source. HPLC, Fourier-transform infrared spectroscopy (FTIR), thermogravimetric analysis (TGA), nuclear magnetic resonance (NMR) spectroscopy, and a 10% mass loss collectively indicated chemical and physical changes in the polymer. Such findings are strongest when supported by appropriate abiotic controls and direct evidence of polymer-derived carbon assimilation or mineralization.

Santo et al. [12] identified a copper-binding laccase from the actinomycete *Rhodococcus ruber* that could oxidize and depolymerize polyethylene (PE) sheets. Incubation with extracellular laccase from copper-induced cells reduced the polymer’s molecular weight by 15–20%, while FTIR detected a carbonyl peak consistent with enzymatic oxidation. Using available genomic information from microorganisms associated with polystyrene (PS) degradation, Hou and Majumder [17] identified alkane hydroxylases, cytochrome P450 enzymes, and monooxygenases as candidate enzyme classes for cleaving carbon–carbon bonds. These hydroxylating enzymes may attack PS side chains and oxidize aromatic structures. The candidate enzymes were distributed across Bacteroidetes, Firmicutes, Actinobacteria, and Proteobacteria, providing targets for further experimental validation. CaPETase and its engineered M9 variant further demonstrate how natural enzyme discovery and rational design can combine activity at ambient temperature with high thermostability [62].

Loredo-Treviño et al. [63] evaluated fungal colonization of polyurethane (PUR) sheets supplied as the sole nutrient source. Twenty-two fungal species from the genera *Aspergillus*, *Trichoderma*, *Paecilomyces*, *Penicillium*, *Alternaria*, and *Fusarium* were screened for their ability to colonize and depolymerize PUR. Urease, protease, esterase, and laccase activities were among the most prominent. Although colonization alone does not demonstrate complete biodegradation, the observed enzyme activities identify promising strains for further studies using complementary chemical and mineralization analyses.

Many microbial strains including *Lysinibacillus xylanilyticus*, *Aspergillus niger*, *Comamonas* sp., *Delftia* sp., *Stenotrophomonas* sp., *Brevibacillus* sp., *Aneurinibacillus* sp., *Stenotrophomonas panacihumi* PA3-2, *Phanerochaete chrysosporium* NCIM 1170, *Rhodococcus rhodochrous* ATCC 29672, *Pestalotiopsis microspora*, and *Pseudomonas stutzeri* have been reported to colonize or depolymerize synthetic polymers [11,14,15,64,65,66,67,68,69] (Table 1). Current research increasingly combines environmental screening with metagenomics, protein engineering, and synthetic biology. Metagenomic mining has identified more than 30,000 putative homologues associated with the degradation of 10 plastic types [70], while experimental work has evaluated the marine fungus *Alternaria alternata* FB1 for PE degradation [71]. Engineered *Chlamydomonas reinhardtii* has also been used to secrete active PHL7 and release terephthalic acid from PET, illustrating a photosynthetic platform for producing plastic-degrading enzymes [72]. Future applications will require rigorous validation of enzyme activity, polymer-derived products, and performance under environmentally or industrially relevant conditions.

### 4.2. Plastic-Eating Invertebrates and the Associated Microbiomes

The use of plastic-eating invertebrates, particularly mealworms and waxworms, has attracted increasing attention. These animals mechanically fragment plastics, while microorganisms in their gastrointestinal tracts may contribute to depolymerization and assimilation. Omnivorous pests such as mealworms harbor adaptable gut communities capable of using diverse substrates, including decaying grain, cereal products, and, under experimental conditions, plastics as a carbon source [75,84,85,86]. On a conventional diet, the posterior gut of mealworms contains microorganisms such as *Enterobacter*, *Spiroplasma*, and *Clostridium*, whereas the anterior gut is dominated by facultative anaerobes including *Pantoea*, *Lactococcus*, and members of Bacillaceae. Larvae of *Tenebrio molitor* remained viable for up to one month while consuming polystyrene foam as their sole carbon and energy source [86]. Using ^13^C-labelled material, researchers reported that at least 50% of ingested polystyrene carbon was converted to CO_2_, with residual polymer detected in frass [86]. Yang et al. [75] isolated *Exiguobacterium* sp. strain YT2 from the larval gut; after four weeks, the bacterium formed biofilms and produced surface corrosion and cavities on polystyrene sheets, reduced molecular weight by up to 11%, decreased hydrophobicity, and introduced polar functional groups. After two months, the strain produced a 7.4 ± 0.4% mass loss and released water-soluble products. Larvae of the lesser waxworm, *Achroia grisella*, have also been reported to complete their life cycle while consuming high-density polyethylene (HDPE). Consumption increased by 1.83 mg per day over eight days, and the polymer lost more than 40% of its mass. FTIR and proton nuclear magnetic resonance (1H NMR) analyses of frass detected unsaturated hydrocarbons and alcohol and carbonyl groups, consistent with the formation of degradation intermediates [87].

Yang et al. [88] reported that larvae of the Indian mealmoth, *Plodia interpunctella*, could degrade polyethylene (PE) films. Two bacterial strains, *Enterobacter asburiae* YT1 and *Bacillus* sp. YP1, were isolated from the larval gut. After each strain was incubated with PE film for one month, atomic force microscopy (AFM) and scanning electron microscopy (SEM) revealed holes and cavities on the polymer surface. X-ray photoelectron spectroscopy (XPS) and micro-attenuated total-reflectance FTIR imaging also detected carbonyl groups. Superworm larvae, *Zophobas atratus*, remained viable for one month while consuming polystyrene foam as their sole carbon and energy source [89]. FTIR, NMR, and gel-permeation chromatography (GPC) indicated depolymerization and the formation of low-molecular-weight products in the larval gut. Antibiotic treatment reduced this activity, supporting the role for the gut microbiota. Zhang et al. [90] reported polyvinyl chloride (PVC) degradation by *Klebsiella* sp. EMBL-1 isolated from the gut of *Spodoptera frugiperda* larvae. Genomic and transcriptomic analyses indicated biofilm formation, secretion of extracellular proteins, and upregulation of genes encoding putative PVC-degrading enzymes. Collectively, these studies suggest that invertebrate gut microbiota may facilitate polymer transformation, but rigorous controls are essential.

Both this claim and a related report that *Galleria mellonella* (waxworm) saliva oxidizes PE under mild conditions [91] have since been directly challenged. Stepnov et al. [92] attempted to reproduce both results using the original protocols and found no evidence of PE or PVC breakdown in either system. The FTIR bands the original studies read as oxidation products, they showed, actually came from residual protein adsorbed onto the plastic surface, not from any chemical change in the polymer itself. What followed is unusual and worth noting; rather than dispute the critique, the authors of the Klebsiella study repeated their own assay and published a formal addendum agreeing they could not reproduce their earlier finding, suggesting the degradation products they had originally detected by GC-MS most likely came from unstable components in their self-prepared PVC powder rather than enzymatic activity [93]. Two high-profile claims, independently checked, did not hold up, and one research group said so in print. That sequence is the clearest illustration in this literature of why a positive FTIR signal is not, by itself, evidence of biodegradation, and why claims of invertebrate-mediated plastic breakdown need the confirmatory methods discussed in Section 5.8 before they are accepted.

The viability of *T. molitor* and *Z. atratus* as biodegradation agents should be judged against what leaves the insect, not only against what disappears from the test polymer. Neither species mineralizes its plastic diet completely: in *T. molitor* fed polystyrene, roughly half of the ingested carbon is converted to CO_2_, while most of the remainder is excreted in frass as oxidized oligomers and sub-micron plastic fragments rather than fully degraded [94,95]. *Z. atratus* shows a comparable pattern. This incomplete mineralization carries an ecological cost: frass containing residual polymer, if applied to soil as an amendment or released into the environment, becomes a new source of microplastics and, on further weathering, nanoplastics, rather than an endpoint for the pollution problem. Proposals to deploy these insects at scale for plastic-waste management therefore require a downstream step, such as composting or further microbial treatment of the frass; ingestion and initial breakdown alone should not be treated as evidence that the plastic has been dealt with. The engineered *Drosophila melanogaster* system in Table 2 depended on heterologous expression of a bacterial PETase gene rather than an enzyme native to the fly, which itself indicates that unmodified flies do not possess endogenous machinery for depolymerizing PET. Whether dipterans more broadly harbor any native capacity to attack pure hydrocarbon-chain polymers such as PE or PP, as opposed to relying on mechanical fragmentation and gut-microbiome cooperation, remains unestablished.

### 4.3. Biodegradable Polymers

The term “bioplastic” is often used ambiguously. It may refer to plastics produced wholly or partly from biomass, plastics that are biodegradable under specified conditions, or both. Bio-based origin does not necessarily imply biodegradability, and some petroleum-derived polymers are biodegradable. Unlike persistent conventional plastics such as PE, PP, and PS, certified biodegradable plastics are designed to undergo biological conversion under defined conditions, such as industrial composting, soil, or aquatic environments. Growing concern about plastic pollution has therefore stimulated research into biodegradable and biocompatible materials that could replace selected petroleum-based products where appropriate.

Biotechnology supports the production and modification of natural and bio-based polymers for use in bioplastic composites. Candidate materials include polyesters, plant-derived resins, cellulose composites, gelatin, collagen, casein, lignin, and starch-based polymers. Their degradability depends on chemical structure, formulation, crystallinity, additives, and environmental conditions. Polylactic acid (PLA) and polyhydroxyalkanoates (PHAs), for example, can be produced at scale through fermentation using microbial cultures and agricultural feedstocks [96]. Under suitable conditions, some biodegradable polymers can be mineralized to CO_2_, H_2_O, and biomass, whereas incomplete degradation or inappropriate disposal may still leave residues. Starch is a widely studied plant-derived polysaccharide because of its structural versatility and capacity to hydrolyse into sugars and organic acids. These products can serve as renewable feedstocks for plastics and biofuels, making starch a versatile component of biodegradable materials [97,98,99].

**Table 2:** Summary of recent studies on the biodegradation of various plastics by invertebrates.

Invertebrate Species	Polymer Type	Reported Evidence	Evidence	Duration	Reference
Indian mealmoth (*Plodia interpunctella*); *Enterobacter asburiae* YT1 and *Bacillus* sp. YP1	Polyethylene (Mw/Mn/crystallinity not reported)	SEM and AFM revealed cavities and holes 0.3–0.4 μm deep; XPS detected carbonyl-group formation	Depolymerization	28 days	[88]
Yellow mealworm (*Tenebrio molitor* Linnaeus)	Polystyrene (Mn = 40,430; Mw = 124,200; PS purity > 98%)	47.7% of ingested carbon converted to CO_2_, based on solid-state NMR analysis of frass	Mineralization (CO_2_ conversion, NMR)	16 days	[86]
Yellow mealworms (*Exiguobacterium* sp. strain YT2)	Polystyrene (molecular weight reported as “lower” post-treatment; no baseline Mw/Mn or crystallinity)	SEM revealed holes and cavities 0.2–0.3 mm wide; 7.4 ± 0.4% weight loss and release of water-soluble products	Depolymerization	60 days	[75]
Dark mealworm (*Tenebrio obscurus*)	Polystyrene (Mn reduction 26.03%)	26.03% decrease in molecular weight; appearance of carbonyl groups detected by FTIR	Depolymerization	31 days	[85]
Darkling beetle (*Plesiophthalmus davidis*)	Polystyrene (Mw/Mn/crystallinity not reported)	34.27 ± 4.04 mg of PS foam ingested per larva with no mortality; a significant C–O peak detected by FTIR	Depolymerization	20 days	[100]
Superworm (*Zophobas atratus*); *Pseudomonas aeruginosa* strain DSM 50071	Low-density polyethylene (Mw/Mn/crystallinity not reported)	Hydroxyl and carbonyl groups detected by NMR; holes observed by SEM; reduced water-contact angle on the polymer surface	Depolymerization	60 days	[16]
Greater wax moth (*Galleria mellonella*); *Lysinibacillus fusiformis*, *Bacillus aryabhattai*, and *Microbacterium oxydans*	Low-density polyethylene (Mw/Mn/crystallinity not reported)	Bacterial growth on the PE surface peaked at 24 h; up to 13.5% weight loss	Surface/fragmentation	18 days	[101]
Red flour beetle (*Tribolium castaneum*)	Polystyrene (Mw/Mn/crystallinity not reported)	12.14 ± 1.4% weight loss; altered thermal properties; aliphatic and aromatic functional groups detected by FTIR	Depolymerization	30 days	[102]
Lesser mealworm (*Alphitobius diaperinus*)	Polystyrene (Mw/Mn/crystallinity not reported)	Isolated bacteria attached to PS surfaces; SEM revealed thin fibrillar structures connecting cells to the abiotic surface	Surface/fragmentation (colonization only)	60 days	[103]
Benthic clamworm (*Perinereis vancaurica*)	Polystyrene (Mw: 216.9 → 212.3 kDa, −2.1%; Mn: 73.5 → 64.3 kDa, −12.5%; PDI 3.3 → 2.95; film purity ≥ 99.9%)	Accumulation of microplastics with a mean diameter of 0.6 ± 0.2 mm in the clamworm gut	Depolymerization	-	[104]
Engineered *Drosophila melanogaster* expressing glycosylated PETase	Poly(ethylene terephthalate) (Mw/Mn/crystallinity not reported)	In vivo hydrolysis of water-soluble PET copolymer and sustained degradation of solid PET under alkaline conditions	Depolymerization	Up to 60 days	[105]

Values are reported as stated in the original source study; where the original study conducted replicate trials, results are given as mean ± SD. Studies that did not report replication are shown as single values.

PLA is a well-established biodegradable polyester that can be processed using techniques such as injection moulding and extrusion. Its versatility has supported applications across the thermoplastics industry [106]. Blends of starch, PLA, and other biodegradable polymers may provide alternatives to selected conventional plastics [107]. This versatility comes with a caveat. PLA is compostable, but chiefly under the aerobic, thermophilic conditions of an industrial composting facility, typically 58 °C and above sustained for weeks; at the mesophilic temperatures found in soil, freshwater, or the marine environment it degrades slowly or not at all, and home composting rarely reaches the required temperature [108]. Microalgae and macroalgae are also being investigated as renewable feedstocks. Torres et al. [109], for example, used residual microalgal biomass to prepare composites with poly(butylene adipate-co-terephthalate) (PBAT) through extrusion and injection moulding. Poly(ε-caprolactone) (PCL) is a biodegradable synthetic polyester produced by ring-opening polymerization of ε-caprolactone and has a melting point of approximately 60 °C. Under favourable laboratory conditions, microbial and enzymatic activity can degrade PCL within relatively short periods [38,110]. *Penicillium* sp. strain 26-1, *Aspergillus* sp. strain ST-01, and *Bacillus licheniformis* have been reported to degrade PCL under anaerobic conditions [111,112]. Poly(β-propiolactone) (PPL), which has a structural unit similar to that of polyhydroxybutyrate (PHB), can be degraded by PHB depolymerases and lipases [113,114] and by microorganisms such as *Variovorax paradoxus* and *Streptomyces* sp. [115].

Despite the promise of biodegradable polymers and biologically mediated degradation, the underlying mechanisms and environmental performance remain incompletely understood. Broader application requires studies that distinguish fragmentation, depolymerization, assimilation, and mineralization; characterize microbial community composition and interactions; and evaluate performance under realistic disposal conditions. Standards-development and certification organizations including ASTM International, the European Committee for Standardization (CEN), the US Composting Council (USCC), and Japan’s GreenPla program have developed frameworks for assessing compostability and biodegradability [9,116]. However, harmonized testing and transparent reporting remain necessary to compare results across materials, laboratories, and environments.

Biodegradability is also not synonymous with environmentally friendly. Earthworm studies show that biodegradable and compostable microplastics can still produce measurable biological or ecotoxicological effects, comparable in some respects to conventional microplastics. Commercial compostable bags degraded at different rates in vermicomposting systems and some fragments proved toxic to *Eisenia andrei* [117]. Biodegradable and compostable polyethylene-based microplastics altered soil ecopathological endpoints in *Eisenia fetida* in ways not clearly distinguishable from conventional high-density polyethylene [118]. A meta-analysis across earthworm studies found that while conventional microplastics primarily reduced survival and growth, biodegradable microplastics carried distinct, not necessarily lower, risks to reproduction and gut microbiota [119]. Reporting that a polymer is biodegradable or compostable therefore does not, on its own, establish that its degradation products or intermediate fragments are biologically inert. Assessing this risk systematically would require standard aquatic or soil ecotoxicology assays; for instance, *Daphnia magna* or zebrafish embryo bioassays that applied directly to the water-soluble fractions released during partial degradation, alongside chemical characterization of the specific breakdown products, rather than inferred from the biodegradability of the parent polymer. To our knowledge, this pairing has rarely been done for the invertebrate- and microbe-mediated pathways.

## 5. Polymer Degradation Determination Methods

Plastic biodegradation can be assessed using microbiological, physical, chemical, and analytical methods. To classify a synthetic polymer as biodegradable, investigators must provide sufficient evidence that biological agents depolymerize, assimilate, and preferably mineralize polymer-derived carbon. No single measurement is generally sufficient; robust assessments combine complementary techniques and appropriate abiotic and biotic controls. Figure 2 summarizes commonly used approaches.

### 5.1. Surface Topography Analysis

Surface-topography analysis evaluates changes such as cracks, cavities, discoloration, fragmentation, roughening, and biofilm formation [11]. Although stereomicroscopy can identify preliminary signs of surface alteration, these observations do not demonstrate that biological metabolism caused polymer degradation. Higher-resolution techniques such as AFM and SEM provide more detailed evidence of changes in surface roughness and morphology. Peixoto et al. [69] used AFM and SEM to examine PE products, focusing on nanoscale roughness and micrometer-scale damage. Mohanrasu et al. [120] combined SEM and FTIR to investigate HDPE and polycyclic aromatic hydrocarbons (PAHs). Nevertheless, erosion or microbial colonization of a plastic surface is not sufficient proof of biodegradation because microorganisms may use the material only as a surface for biofilm formation [121]. Surface imaging should therefore be combined with chemical, molecular-weight, product, or mineralization analyses. Recent methodological studies also warn that FTIR-band changes may arise from proteins, lipids, additives, or other contaminants and should not be interpreted as stand-alone evidence of polymer oxidation [92,122].

### 5.2. Functional Groups of Surface Properties

Changes in functional groups on plastic surfaces are commonly analysed using FTIR. The technique can detect alterations in hydrogen bonding, crosslinking, oxidation, and other chemical features of solid or liquid polymers [123,124]. Depending on the polymer and degradation pathway, microbial activity may alter carbonyl, hydroxyl, vinyl, or ester indices; however, the direction and magnitude of these changes are not universal and must be interpreted against appropriate controls. Harshvardhan and Jha [125] screened 60 marine bacterial isolates for their ability to degrade LDPE. Changes in vinyl-bond, carbonyl-bond, and ester-carbonyl indices were observed for *Bacillus subtilis* H1584, *B. pumilus* M27, and *Kocuria palustris* M16. FTIR can therefore provide valuable evidence of chemical changes, particularly because oxidation can increase surface hydrophilicity and susceptibility to microbial attack [20,43]. Nevertheless, spectroscopy should be paired with measurements of molecular-weight reduction, degradation products, assimilation, or mineralization. A carbonyl index calculated from a raw spectrum is not, by itself, proof of polymer oxidation. The carbonyl-associated region overlaps with the amide I and amide II bands of proteins, centered near 1650 cm^−1^ and 1545 cm^−1^, and these bands are frequently far stronger than any oxidation signal from the polymer itself. Gut tissue, silk, pollen, and, most relevantly for invertebrate assays, microbial biofilm attached to the plastic surface can all contribute amide absorbance in this region, inflating the apparent carbonyl index without oxidation of the polymer backbone having occurred [122]. A reported increase in carbonyl index should therefore be accompanied by a baseline spectrum of the relevant biological material, or by a complementary method not vulnerable to the same interference, such as those discussed in Section 5.8.

### 5.3. Weight Loss Determination

Gravimetric mass loss is among the most widely used measurements in laboratory and field studies of polymer degradation [126]. However, it does not directly demonstrate biodegradation. For formulations containing substantial quantities of additives, such as PVC, mass loss may reflect additive leaching rather than cleavage of the polymer backbone. Residual biofilms, incomplete cleaning, hydrolysis, and loss of fragmented particles can also bias measurements. Samples should therefore be cleaned using a validated procedure, dried to constant mass, and compared with appropriate abiotic controls. Polymer properties, including crystallinity, molecular weight, and additive composition should be characterized and reported because commercially available research plastics can differ substantially [127]. Mass loss is best treated as a screening indicator and combined with techniques such as molar-mass analysis, product identification, radiolabelling, or respirometry to demonstrate polymer transformation and mineralization.

### 5.4. Radiolabelling

Radiolabelling is a sensitive technique in which carbon isotopes are incorporated into the polymer and traced during microbial growth [128]. Mineralization can then be quantified from the production of gases such as CO_2_ or CH_4_ [129]. Although radiolabelling provides strong evidence that gaseous products originate from the polymer, it is costly, requires specialized facilities and trained personnel, and creates radioactive-waste management obligations. These constraints have limited its use relative to more accessible methods such as FTIR and SEM. Stable-isotope probing with ^13^C-labelled PE offers a non-radioactive alternative and has been used to quantify the transfer of polymer-derived carbon into CO_2_ and microbial lipids, thereby providing specific evidence of mineralization and assimilation [83].

### 5.5. O_2_ Consumption/CO_2_ Evolution

Aerobic respirometry is a standard approach for evaluating polymer biodegradation. Polymer samples are incubated with microorganisms in the presence of oxygen, and biological oxidation of polymer-derived carbon is assessed by measuring O_2_ consumption or CO_2_ evolution [41,130,131]. Experiments are conducted in controlled systems such as sealed respirometers, with suitable blanks and reference materials. Biodegradation is commonly expressed by comparing measured CO_2_ evolution with the theoretical maximum CO_2_ yield (ThCO_2_). Because background respiration can be substantial and plastic degradation is often slow, accurate airflow measurement, sensitive detection, and long-term monitoring are essential.

CO_2_ generated during biodegradation may also be trapped in barium hydroxide (Ba(OH)_2_) and quantified by titration [75]. Despite its accuracy, long-term respirometry is vulnerable to systematic and operator-related errors when O_2_ consumption and CO_2_ production are close to detection limits. Pagga et al. [132] addressed these challenges by extending tests to six months, increasing nutrient and buffer availability, and constructing a carbon balance. Their work contributed to standardized approaches under ISO 9408 [133] and ISO 9439 [134].. A closed respirometer was used to evaluate a poly(ε-caprolactone)–starch blend, an aliphatic–aromatic copolyester, and microcrystalline cellulose as a reference material, with CO_2_ trapped in a basic solution and quantified through continuous titration and dissolved-carbon monitoring.

### 5.6. Clear-Zone Formation

Clear-zone analysis is a simple, semiquantitative method for screening potential polymer-degrading microorganisms. Fine polymer particles are dispersed in agar, and a clear halo around a microbial colony indicates solubilization or enzymatic hydrolysis of the substrate. Because halo formation does not by itself establish assimilation or mineralization, the method is best used as an initial screen followed by confirmatory analyses [121]. Arefian et al. [135] used clear-zone assays to identify four landfill-derived fungal genera such as *Fusarium*, *Penicillium*, *Ulocladium*, and *Chrysosporium* with potential to degrade polycarbonate. Cho et al. [136] and Zhang et al. [137] similarly used the method to screen *Bacillus licheniformis* and *Bacillus amyloliquefaciens* strains HK1, GSDM02, and GSDM15 for activity against polymeric substrates such as polyvinyl alcohol (PVA).

Clear-zone screening is particularly useful when many isolates must be evaluated. It has been used to identify candidates such as *Aspergillus niger* and *Aspergillus flavus* for subsequent polymer-biodegradation studies [138]. Promising isolates should then be assessed using quantitative and orthogonal methods that distinguish substrate hydrolysis from complete biodegradation.

### 5.7. Biofilms and Microbial Adhesion

Surface hydrophilicity strongly influences microbial attachment and biofilm formation on synthetic polymers [139]. Viability on plastic surfaces can be assessed using methods such as the triphenyl tetrazolium chloride (TTC) reduction assay, while contact-angle and water-uptake measurements characterize changes in surface wettability. More hydrophilic surfaces generally exhibit smaller water-contact angles. Sudhakar et al. [140] incubated LDPE and HDPE with the marine bacteria *Bacillus sphericus* and *B. cereus* for one year, using the plastics as the sole carbon sources. Reduced water-contact angles after incubation indicated increased surface hydrophilicity. Roy et al. [4] likewise reported a decrease in the contact angle of LDPE film from 99.6 ± 3.5° to 92.5 ± 3.5° following incubation with *B. pumilus*, *Bacillus halodenitrificans*, and *B. cereus*. Figure 3 illustrates bacterial attachment and biofilm formation on a polymer surface by a strain isolated in our laboratory from the gut of the mealworm *Tenebrio molitor*; this attachment reflects colonization rather than confirmed degradation of the underlying polymer. These observations support colonization and surface modification, but they should be combined with chemical or mineralization evidence before biodegradation is concluded.

These micrographs are original, unpublished data generated in the authors’ own laboratory. They are included here, with full methodological disclosure, to directly illustrate the distinction this review draws throughout Section 5 between colonization/surface-attachment evidence and confirmed polymer degradation, a distinction the published imagery cited elsewhere in this review does not consistently make explicit, since each source image is framed around its own study’s claims rather than this review’s evidentiary framework. Gut bacteria were isolated from *Tenebrio molitor* reared on polystyrene: worms were surface-disinfected in 75% ethanol, homogenized, and the homogenate was serially diluted and plated on Luria–Bertani agar; colonies with distinct morphology were subcultured to purity and identified by Gram staining. Pure isolates were grown to suspension and inoculated into a carbon-free medium containing polystyrene film as the sole carbon source, alongside two controls processed identically but without polymer or without bacterial inoculation. Cultures were incubated on a rotary shaker at 37 °C for 60 days. Recovered films were rinsed in ethanol and deionized water to remove loosely adherent cells, air-dried, and imaged by field-emission SEM at 998× magnification. No chemical or gravimetric analysis was performed on these samples following biofilm removal, and no claim of polymer degradation is made from this dataset.

Numerous analytical approaches have been used to evaluate the biodegradation of synthetic polymers. However, protocols differ substantially in substrate preparation, controls, incubation conditions, cleaning procedures, analytical thresholds, and reporting. These differences limit comparison across studies and can lead to overinterpretation of surface changes or mass loss. Internationally harmonized methods are therefore needed to measure biodegradation rates under clearly defined environmental conditions. Recent replication and substrate-characterization studies support an evidence framework that combines gravimetry, spectroscopy, molar-mass analysis, degradation-product identification, and isotope tracing with well-characterized polymers and appropriate additive-free, abiotic, and biotic controls [83,92,127].

### 5.8. Carbon Stoichiometry and Isotopic Verification

Every method described above: surface imaging, functional-group analysis, weight loss, clear-zone formation, and biofilm assays detects a change consistent with biodegradation rather than proving it. Surface erosion can result from biofilm attachment without any carbon leaving the polymer. A rising carbonyl index can reflect protein contamination rather than oxidation (Section 5.2). Weight loss can reflect additive leaching rather than polymer breakdown (Section 5.3). What distinguishes genuine mineralization from these forms of surface or analytical artifact is carbon fate, specifically, whether the polymer’s carbon has actually left the material. Two approaches answer this. The first is closed respirometry, in which CO_2_ evolved from a sealed test system is measured against the theoretical CO_2_ yield of the polymer (ThCO_2_); a result close to the theoretical maximum indicates that carbon has left the polymer and entered the mineralization pathway, not merely that the surface has changed. The second is isotope tracing, using ^13^C- or ^14^C-labelled polymer to follow carbon directly into CO_2_ and microbial biomass; stable-isotope probing of polyethylene has demonstrated this approach, quantifying carbon transfer with a specificity that surface methods cannot match [83]. Neither method is a routine addition to a degradation assay. Both are, at present, the evidence that unambiguously separates true biodegradation from the physical or chemical modification of a plastic surface, and the evidence framework this review proposes rests on that distinction.

## 6. Future Challenges and Perspectives

Evidence for polymer biodegradation is increasing, but the literature remains heterogeneous and, for many conventional plastics, inconclusive. Biodegradation involves complex biochemical and ecological interactions that are difficult to reproduce under laboratory conditions. Studies often rely on a single microbial strain, whereas natural degradation may depend on consortia whose members perform complementary reactions. Long incubation periods and low reaction rates further complicate experimental design. Future work should therefore connect functional genes, enzyme activities, community interactions, polymer chemistry, and measured carbon flux to explain how specific polymers are transformed under realistic conditions.

Culture-dependent methods also fail to capture many uncultivated microorganisms. Analytical platforms such as HPLC and gas chromatography–mass spectrometry (GC-MS) can identify and quantify known products, but untargeted metabolomics and high-resolution mass spectrometry may be required to discover unknown intermediates. Future studies should integrate cultivation, next-generation sequencing (NGS), metagenomics, transcriptomics, proteomics, and metabolomics with rigorous polymer analytics. Emerging workflows already combine metagenomic or structure-based mining, protein language models, and experimental validation. VenusMine, for example, identified 14 active PETase candidates, including the thermostable KbPETase [80], while computational engineering of PHL7 variants improved PET conversion under high-substrate conditions [81].

Scale-up remains the field’s weakest link. Most of the results summarized above come from gram-scale batch trials using hand-picked substrates, not from waste streams contaminated with additives, mixed polymers, and biological debris. Ahmad et al. [94] estimated that treating plastic waste at the ton scale with mealworms would require tens of millions of larvae working continuously, a throughput current insect-rearing infrastructure cannot support. Enzymatic systems face a related problem: the high-solids conversion rates reported for TurboPETase and PHL7 variants [55,56,81] have not yet been matched with the carbon mass balances needed to confirm performance once feedstocks are impure and reactors run continuously, rather than the well-defined substrates used in laboratory trials.

Overall, biological approaches could complement reduction, reuse, mechanical recycling, and chemical recycling, but they should not be presented as a stand-alone solution to plastic pollution [141]. Progress will depend on reproducible evidence of depolymerization, assimilation, and mineralization; transparent reporting of polymer composition and test conditions; standardized protocols; and realistic assessments of scalability, energy use, biosafety, and environmental impact. These priorities will help distinguish promising laboratory observations from technologies capable of delivering measurable benefits in waste-management systems.

## 7. Conclusions

This review synthesized current evidence on microbial, enzymatic, and invertebrate-mediated biodegradation of synthetic plastics, alongside the biodegradable-polymer alternatives and analytical methods used to verify degradation. The evidence base is strongest for depolymerization and surface modification, but direct proof of mineralization and carbon assimilation remains rare and is concentrated in a small number of isotope-tracing studies. Reported degradation rates vary widely across studies due to inconsistent reporting of polymer molecular weight, crystallinity, and test duration, and no internationally harmonized standard currently exists for validating plastic biodegradability claims. Closing this evidence gap will require wider adoption of isotope-based mineralization assays, standardized reporting of polymer characteristics, and consortium- or scale-relevant trials rather than single-strain, gram-scale experiments. With these improvements, biological approaches can be positioned as one credible, evidence-based component of an integrated plastic-waste management strategy.

## Figures and Tables

**Figure 1:**
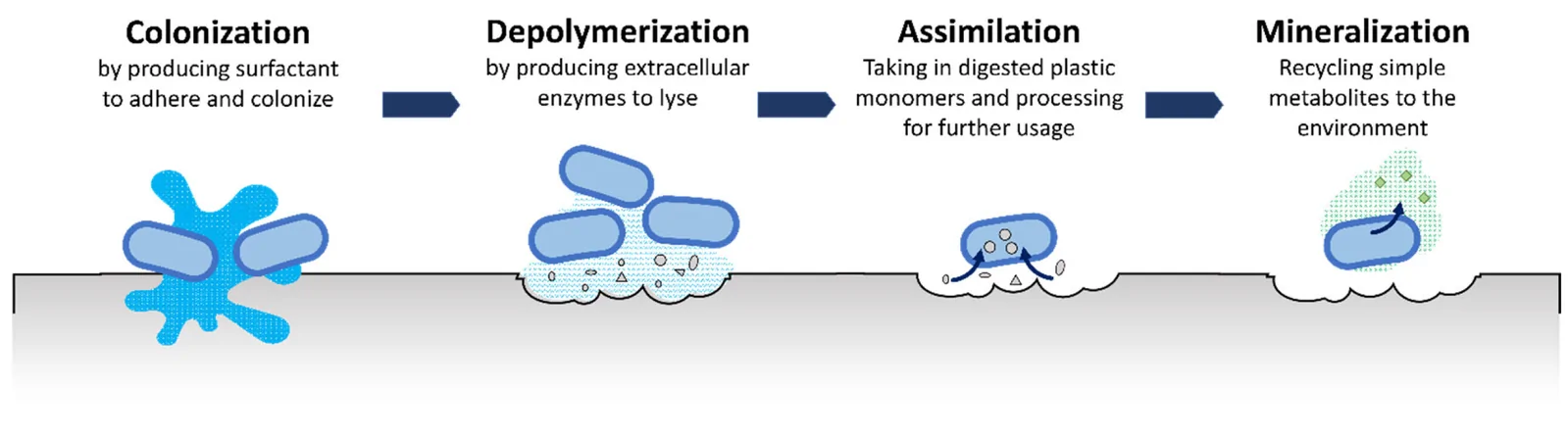
General biodegradation mechanism pathways of a polymer: an overview of how the action of microorganisms can play a role in the degradation of plastic waste.

**Figure 2:**
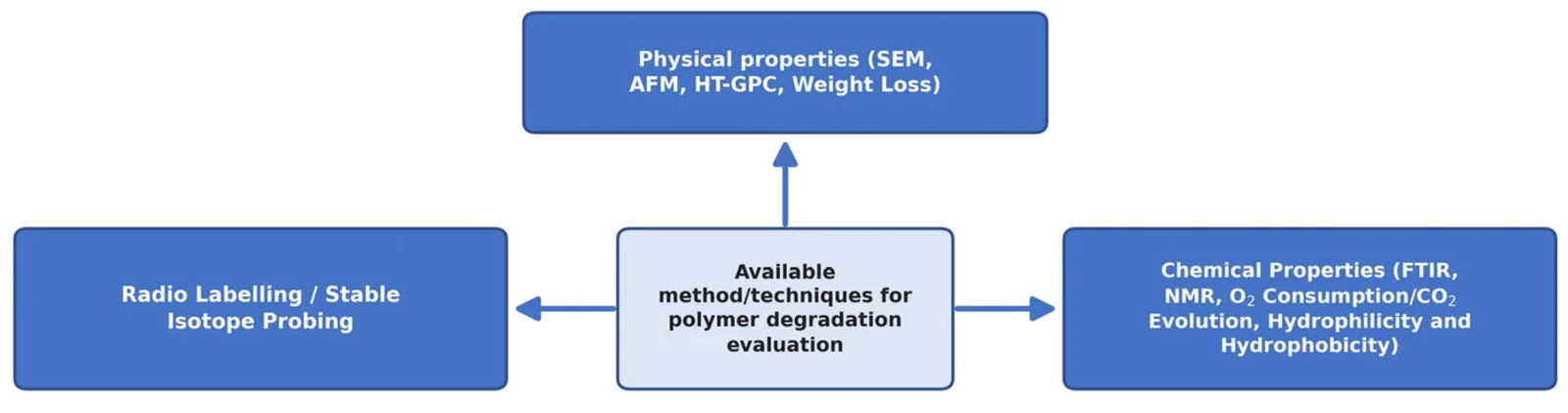
Proposed techniques for polymer degradation evaluation.

**Figure 3:**
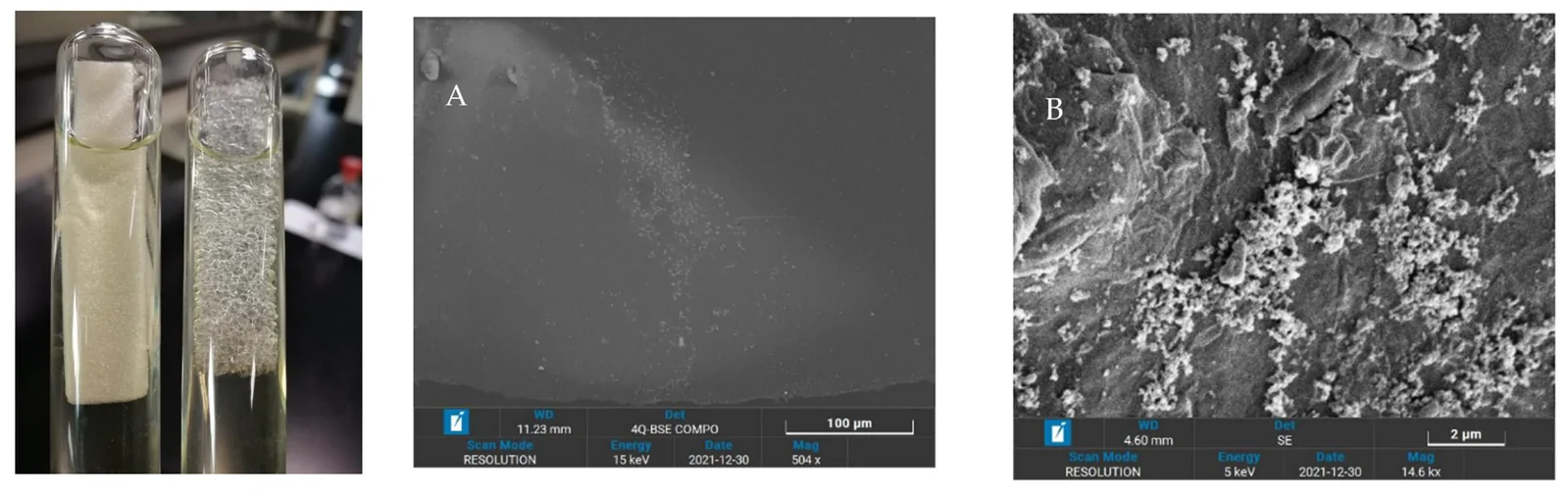
Representative PS samples and scanning electron micrographs of an uninoculated control (**A**) and a surface colonized by gut bacteria (**B**) isolated from *Tenebrio molitor* after incubation for 30 days (photographs by K.-S. Phang). Images were captured at 998× magnification; scale bars are shown at the base of each panel. Panel (**B**) shows extensive biofilm attachment relative to the uninoculated control; this attachment indicates colonization of the polymer surface and is not, by itself, evidence of pitting, cavitation, or degradation of the underlying polymer matrix, which was not independently assessed following biofilm removal in this preliminary dataset.

**Table 1:** Recent studies on plastic biodegradation by microbes.

Microbial Species and Strain	Polymer Type	Reported Evidence	Evidence	Duration	Reference
*Bacillus* sp.	Polystyrene (HIPS—impact-modified grade; Mw/Mn/crystallinity not reported)	23% weight loss	Surface/fragmentation	30 days	[61]
*Comamonas* sp., *Delftia* sp., and *Stenotrophomonas* sp.	Polyethylene (Mw ≈ 191,000 g/mol; unpretreated, no additives; crystallinity loss confirmed but no baseline %)	FTIR (oxidation, vinylene formation, and chain cleavage); changes in surface topography	Depolymerization	90 days	[69]
*Brevibacillus borstelensis*, *Bacillus cereus*	Low-density polyethylene (Mw/Mn/crystallinity not reported)	Weight loss (36%); functional-group changes (aldehyde, ether, and carboxyl groups)	Depolymerization	42 days	[14]
*Aneurinibacillus aneurinilyticus*, *Brevibacillus brevis*, *Brevibacillus agri*	Low- and high-density polyethylene, polypropylene (Mw/Mn/crystallinity not reported)	Weight loss (58%, 46%, and 56%); changes in surface topography; new absorption bands	Depolymerization	140 days	[15]
*Saccharomonospora viridis*	Poly(ethylene terephthalate) (Mw/Mn/crystallinity not reported)	Polymer-film fragmentation; 65% weight loss	Surface/fragmentation		[73]
*Ideonella sakaiensis* (Aromatic polyesterase)	Poly(ethylene terephthalate) (crystallinity change tracked by the source study; starting % not confirmed from available sources)	SEM (topographical changes), substrate docking, and crystallization	Surface/fragmentation	4 days	[74]
*Exiguobacterium* sp. strain YT2	Polystyrene (molecular weight reported as “lower” post-treatment; no baseline Mw/Mn or crystallinity)	SEM (pits and cavities); reduced hydrophobicity; formation of C–O polar groups	Depolymerization	28 days	[75]
*Pseudomonas citronellolis*, *Bacillus flexus*	Polyvinyl chloride (Mn reduced ~10%, GPC; starting Mn not reported)	10% reduction in average molecular weight; 19% weight loss; lower thermal stability	Depolymerization	45 days	[76]
*Penicillium variabile*	Polystyrene (two Mw grades of PS compared—lower Mw mineralized faster)	SEM (topographical changes); FTIR (carbonyl-group formation); GPC (decreased molecular weight)	Depolymerization	7 days	[77]
*Aspergillus clavatus*	Low-density polyethylene (Mw/Mn/crystallinity not reported)	35% weight loss; CO_2_ production measured using the Sturm test; SEM (topographical changes)	Mineralization (Sturm test CO_2_)	90 days	[78]
*Paenibacillus alvei*, *Paenibacillus motobuensis*, *Bacillus velezensis*, *Bacillus pseudomycoides*	Micro-sized polyethylene (Mw/Mn/crystallinity not reported)	14.7% gravimetric weight loss; 22.8% reduction in mean PE-particle diameter	Surface/fragmentation	60 days	[79]
Bacterium HR29-derived TurboPETase	Poly(ethylene terephthalate) (post-consumer PET; starting crystallinity ~32.5%; Mw/Mn tracked over time)	Nearly complete depolymerization at 200 g/kg solid loading	Depolymerization	0.3 days	[55]
KbPETase from *Kibdelosporangium banguiense*	Poly(ethylene terephthalate) (no enzyme thermostability data)	High thermostability and catalytic efficiency; PET monomer release quantified by UPLC	Depolymerization	3 days	[80]
Engineered PHL7-R4 variants	Poly(ethylene terephthalate) (high-solid loading 10–20% *w*/*w* noted; specific starting crystallinity not confirmed)	75–78% conversion of 10% (*w*/*w*) PET; outperformed ICCG	Depolymerization	24 h	[81]
Engineered LCC variant “ICCG”	PET (post-consumer, amorphized/micronized)	90% conversion within 10 h; productivity 16.7 g terephthalate/L/h	Depolymerization	0.4 days (10 h)	[82]
*Rhodococcus ruber* strain C208	Polyethylene (^13^C-labelled)	^13^C tracked directly into CO_2_ and microbial biomass, confirming true mineralization rather than surface change	Mineralization (isotope-confirmed)	30 days	[83]

Values are reported as stated in the original source study; where the original study conducted replicate trials, results are given as mean ± SD. Studies that did not report replication are shown as single values.

## Data Availability

No new data were created or analyzed in this study. Data sharing is not applicable to this article.

## Abbreviations

The following abbreviations are used in this manuscript:

PS	Polystyrene
PE	Polyethylene
PUR	Polyurethane
PP	Polypropylene
LDPE	Low-density polyethylene
HDPE	High-density polyethylene
LMWPE	Low-molecular-weight polyethylene
HIPS	High-impact polystyrene
PVC	Polyvinyl chloride
AFM	Atomic force microscopy
SEM	Scanning electron microscopy
XPS	X-ray photoelectron spectroscopy
FTIR	Fourier-transform infrared spectroscopy
H-NMR	Proton nuclear magnetic resonance
HPLC	High-performance liquid chromatography
TGA	Thermogravimetric analysis
GPC	Gel permeation chromatography
GC-MS	Gas chromatography–mass spectrometry
NGS	Next-generation sequencing
PLA	Polylactic acid
PHA	Polyhydroxyalkanoates
PHB	Polyhydroxybutyrate
PBAT	Poly(butylene adipate-co-terephthalate)
PCL	Poly(ε-caprolactone)
PPL	Poly(β-propiolactone)
BPA	Bisphenol-A
PCB	Polychlorinated biphenyls
RMB	Residual microalgae biomass
PAH	Polycyclic aromatic hydrocarbons
TTC	Triphenyl tetrazolium chloride
ThCO_2_	Theoretical CO_2_ (emissions)
ASTM	American Society for Testing and Materials
USCC	US Composting Council
CEN	European Committee for Standardization
